# The bibliometric and altmetric analysis of chronic traumatic encephalopathy research: how great is the impact?

**DOI:** 10.3389/fneur.2024.1294125

**Published:** 2024-02-08

**Authors:** Lulu Guan, Jingwang Tan, Bote Qi, Yukang Chen, Enyu Tong, Jingcheng Pan, Yu Zou

**Affiliations:** ^1^Department of Sport and Exercise Science, College of Education, Zhejiang University, Hangzhou, China; ^2^School of Public Health, Hangzhou Normal University, Hangzhou, China; ^3^College of Physical Education, Guizhou University of Finance and Economics, Guiyang, China

**Keywords:** chronic traumatic encephalopathy, altmetric attention score, bibliometric analysis, comparation, twitter

## Abstract

**Background:**

The study of chronic traumatic encephalopathy (CTE) has received great attention from academia and the general public. This study aims to analyze the research productivity on CTE and investigate the most discussed articles in academia and the general public by conducting bibliometric and altmetric analyses.

**Methods:**

Data of articles were obtained from the Web of Science Core Databases and Altmetric Explore. VOSviewer and CiteSpace software were used to analyze and visualize the articles. The correlation between Altmetric attention scores (AAS) and citation counts were assessed by Spearman correlation coefficient.

**Results:**

788 publications of CTE were eventually gathered and analyzed, and 100 articles with highest citation counts (Top-cited) and 100 articles with highest AASs (Top-AAS) were then identified. The keywords density map showed both the general public and the scientists were particularly interested in the risk factors and pathology of CTE, and scientists were interested in the causes and characteristics of neurodegenerative diseases while the public became increasingly concerned about the detection and prevention of CTE. By examining the shared characteristics of the 44 articles (High-High articles) that overlapped between Top-cited and Top-AAS articles, we identified certain traits that may potentially contribute to their high citation rates and high AASs. Besides, significant positive correlations with varied strength between AAS and citation were observed in the 788 articles, Top-cited, Top-AAS and High-High datasets.

**Conclusion:**

This study is the first to link bibliometric and altmetric analyses for CTE publications, which may provide deeper understanding of the attention of the scientists and the general public pay to the study of CTE, and offer some guidance and inspiration for future CTE in the selection of research topics and directions.

## Introduction

Chronic traumatic encephalopathy (CTE) is a progressive tauopathy that occurs due to repetitive mild traumatic brain injury (1). Over 93 years ago, Martland originally described the clinical aspects of a progressive neurological decline as “punch drunk,” which occurred after repetitive brain trauma in boxers (2). With the development of research, “chronic traumatic encephalopathy” was widely used to describe the disease. Later studies reported that CTE was unrestricted to boxing but also happened in various contact sports, including football, wrestling, rugby, hockey, lacrosse, soccer, and skiing (1). More than that, CTE has been diagnosed in military veterans with combat exposure and others who have suffered frequent head impacts (3–5). Although some reports suggest that degenerative brain disease is almost unavoidable for athletes participating in certain sports, a cause-and-effect relationship between exposure to contact sports and CTE has not been demonstrated (6). Yet, the actual prevalence of CTE is unknown (7). There are currently no definitive criteria for a diagnosis of CTE during life, and identifying CTE in a living individual is still challenging. The disease can only be definitively diagnosed through post-mortem examination of the brain tissue (8). In 2014, based on the literature of the clinical manifestations of CTE from 202 published cases, research diagnostic criteria for traumatic encephalopathy syndrome (TES) were proposed to diagnose CTE pathology in life (9). These criteria were used as a starting point and initial organizing structure for the development of following consensus criteria- NINDS Consensus Diagnostic Criteria for TES (9). And these criteria would be an invaluable tool to enhance the ability to diagnose and manage TES/CTE in individuals and possibly mitigate the associated consequences. Meanwhile, clinical symptoms of CTE can be nonspecific, and there may be overlap with other neurodegenerative diseases (10). In recent years, significant progress and achievements have been made in the field of CTE research, such as the development of advanced imaging techniques (11), identification of specific protein biomarkers (12), underlying pathogenesis of CTE (13), and potential therapeutic targets and interventions (14). In addition to the attention received in the research field, the term CTE has gained significant public attention due to intense media coverage. It has even been the subject of numerous documentaries and a Hollywood movie. Since CTE poses a threat to a significant part of the population and attracts the attention of scientists and the general public, it is important to analyze the dissemination and influence of CTE-related research in academia and the general public.

Bibliometrics is a traditional useful tool for evaluating the productivity, impact, and research trends in a certain field. From a scientific perspective, bibliometrics for determining the value of scientific research centers on the number of times the article is cited and the impact factor of the journal in which the article is published (15, 16). Besides, with the emergence and development of the internet and social media, altmetrics, shorter for alternative metrics, are increasingly utilized as nontraditional metrics of scholarly impact by a weighted calculation of the attention an article receives online (17). Data resources for altmetrics include Twitter, Facebook, blogs, news outlets, Wikipedia, Google, Weibo, Reddit, and other online platforms such as YouTube (18). Altmetric Explorer (Altmetric LLP, London, United Kingdom) generates a weighted score, known as Altmetric Attention Score (AAS). AAS tracks the online presence of articles by measuring and compiling the mentions an article receives across various social media outlets. Besides, AAS allows for continuous updating, and reflects an article’s online attention trends. In recent years, CTE has garnered increased public attention. The most-read article with an extremely AAS published in *The Journal of the American Medical Association (JAMA)* in 2017 reported that 110 of 111 (99%) former football players were suspected of having CTE (19). The article caused extensive controversy and sustained attention and discussion in society until now. Thus, altmetric analysis of CTE productions can help us understand how CTE research is being received and utilized by the general public, and provide evidence of the broader impact of CTE research beyond traditional bibliometric metrics.

Studies have combined bibliometric and altmetric analyses to conduct in-depth researches in some research areas, including osteoporosis, nuclear medicine, reproductive biology, obstetrics and gynecology, and urology (20–24). There were some bibliometric or altmetric studies on CTE in the literature (25–27). However, to the best of our knowledge, this is the first study that combines bibliometric with altmetric analysis about CTE. In this study, we aim to address the following issues: 1. identify the characteristics of total CTE article; 2. examine the traits and commonalities/differences between articles with the highest citation counts and articles with the highest AASs; 3. determine if there is any overlap between articles with the highest citation counts and articles with the highest AASs; 4. conduct an analysis of these overlapped articles. This study may be beneficial to the publication of papers by investigators and the design for the future research of CTE.

## Materials and methods

### Data sources

The data for measurement and statistical analysis were screened from the Web of Science Core Collection (WoSCC) and Altmetric Explorer^1^ on March 2023. We chose WoSCC because it collects scientific publications with the most significant impact and is used as the main criterion in academic decision making. Altmetric Explorer was used to obtain AAS data of the literature from WoSCC. Approval by institutional review boards was not required as the analysis was based on publicly accessible data. This cross-sectional altmetric and bibliometric study followed the Strengthening the Reporting of Observational Studies in Epidemiology (STROBE) reporting guideline for cross-sectional studies (28).

### Search strategy

The search strategy in the WoSCC database were as follows:

#1 TI = “chronic traumatic encephalopathy.”

#2 AB = “chronic traumatic encephalopathy.”

#3 AK = “chronic traumatic encephalopathy.”

#4 #1 OR #2 OR #3.

### Inclusion and exclusion criteria

*Inclusion criteria* Document type: articles and reviews; Language: English. The results were arranged using a link on WoSCC database system “sort-by Citation-highest first.” Then, three independent investigators reviewed the titles and abstracts and deleted studies that were not associated with CTE. Any disagreements were resolved through discussion, and the input of an independent colleague would be sought where consensus could not be reached. The 839 records were selected and exported to an excel format. The DOI or PubMed identification number of articles were entered into the advanced search tool of Altmetric Explorer.

**Figure 1:**
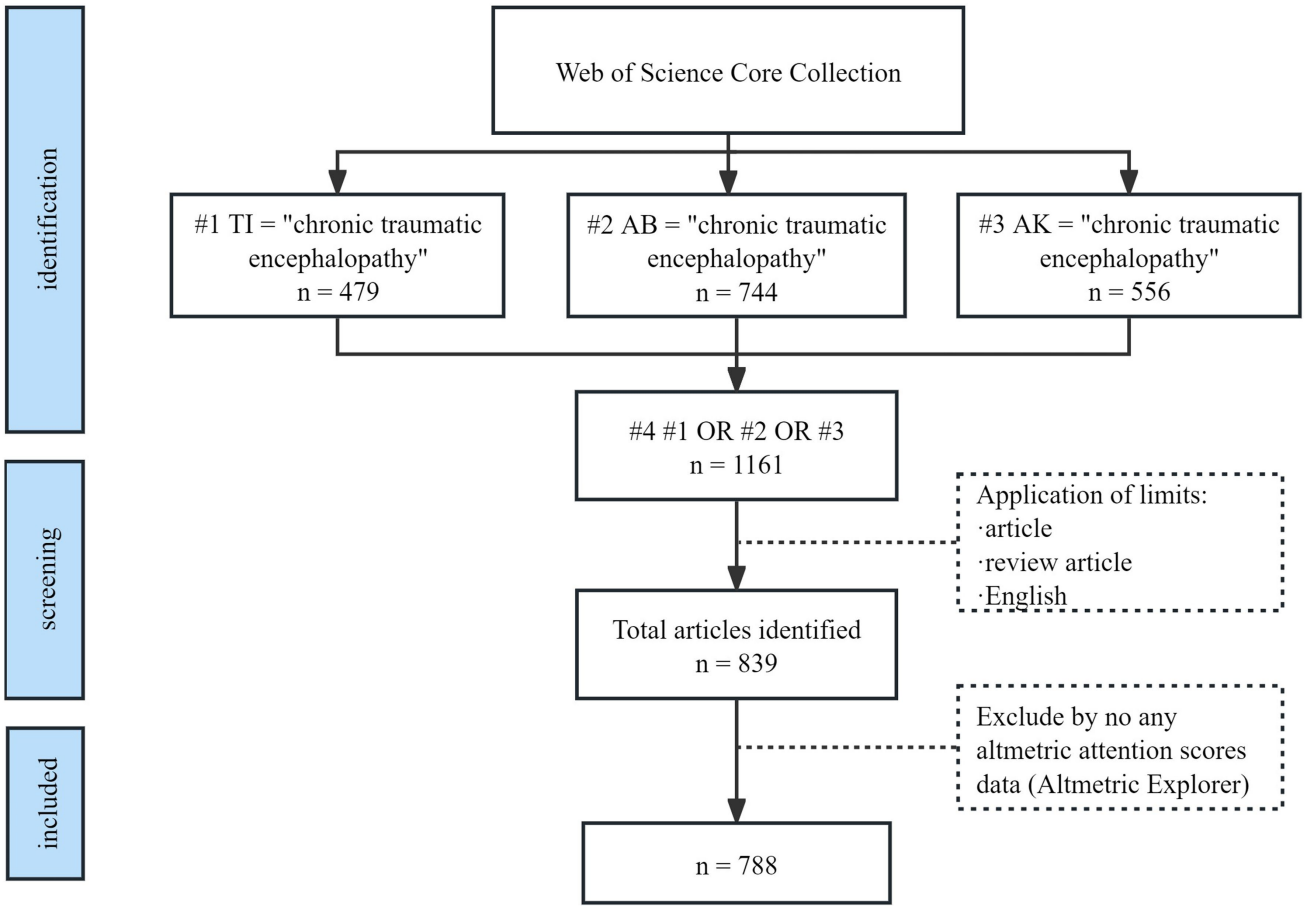
Flowchart detailing the paper collection and screening process.

*Exclusion criteria* Articles without any AAS data were excluded. Eventually records for 788 articles that received altmetric activities were retrieved (Figure 1).

### Analysis of articles

Bibliometric data of 788 articles were downloaded from the WoSCC, and altmetric data of 788 articles were downloaded from Altmetric Explorer on March 2023. The analyses were performed using IBM SPSS, version 24 (Statistical Package for Social Sciences, Chicago, IL). For bibliometric data, we recorded title, study type, topic of the study, authors, organizations, publication year, citation number, keywords, journal name and impact factor. For altmetric data of 788 articles, we recorded the AASs and the number of mentions on Twitter, news outlets, blog posts, policy mentions, patent mentions, peer mentions, Facebook, and Wikipedia for each article. To provide insight into the characteristics of CTE research that with high citation rates and high AASs, the 100 articles with highest citation counts (Top-cited) and the 100 articles with highest AASs (Top-AAS) were identified for further research. The Spearman correlation coefficient was used to assess the correlation between AASs and citation numbers, and was interpreted according to r-level as follows: <0.19 (very weak), 0.2–0.39 (weak), 0.4–0.59 (moderate), 0.6–0.79 (strong), and > 0.8 (very strong). *p* < 0.01 was considered statistically significant.

### Data visualization

In this study, VOSviewer (version 1.6.18) and CiteSpace (version 6.1.R2) were used for data visualization of Top-cited and Top-AAS articles. The keyword density visualization map was plotted by utilizing VOSviewer to identify the study subjects in a certain field and explore the research hotspots (29). The timeline review of keywords was exported by CiteSpace to show the chronological distribution and historical evolvement of knowledge domains (30).

## Results

### Characteristics of total 788 articles

**Figure 2:**
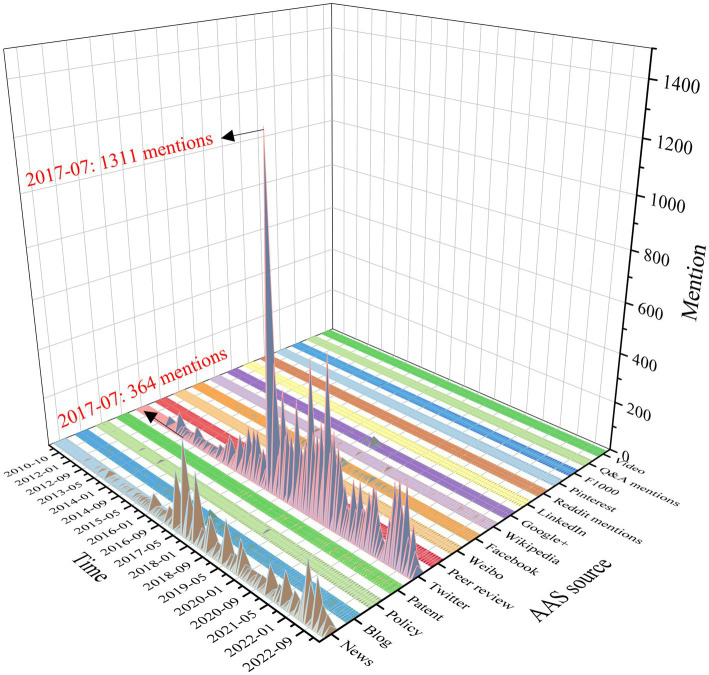
Mentions over time of each AAS online source of 788 research outputs. The peak of News and Twitter appeared in July 2017 with 364 and 1,311 mentions.

Based on the study’s strategic flowchart, we eventually gathered 788 publications, and the related data were downloaded from WoSCC and Altmetric Explorer. As shown in Table 1, among the 788 articles, 63 articles were cited 0 times, 717 were cited 1 to 499 times, 5 were cited 500 to 999 times, and 3 were cited ≥1,000 times. There were 30 articles with AASs of 0, 741 articles with AASs between 1 and 499, 12 articles with AASs between 500 to 999, and 5 articles with AASs of ≥1,000. There was a moderate correlation between AASs and citation numbers (*r* = 0.406**, *p* < 0.01). We then analyzed the average number of online mentions per source for the 788 articles included in the study. Each online source in altmetrics is assigned a weight, such as 8 for every mention in a news article (Supplementary Table S1), and each publication is assigned every source score. The results showed that for the 788 articles the average score of Twitter was 24.74 and the average score of News was 6.40, which were far higher than other sources, indicating that Twitter and News outlets were the principal drivers of attention (Table 1). To provide a more detailed analysis of the AASs, we tracked the sources of AASs for 788 articles over time. Figure 2 showed the temporal evolution of mentions for all 788 research outputs between 2009 to 2023. The highest point of the waterfall chart, representing the peak of attention, manifested on Twitter in July 2017 with 1,311 mentions. The peak of News also appeared in July 2017 with 364 mentions. This surge in attention was not arbitrary, but directly correlated with the publication of a groundbreaking article by Dr. McKee et al. titled “Clinicopathological Evaluation of Chronic Traumatic Encephalopathy in Players of American Football,” published in *JAMA* (19). This work presented the largest CTE case series until 2017, revealing the presence of CTE in all but one of the 111 (99%) participants who were former National Football League players.

**Table 1:** Characteristics of total 788 articles.

Characteristics		Results
Citation	Citation ≥1,000	*n* = 3
	Citation 500–999	*n* = 5
	Citation 1–499	*n* = 717
	Citation 0	*n* = 63
	Average citation	48.85
AAS	AAS ≥1,000	*n* = 5
	AAS 500–999	*n* = 12
	AAS 1–499	*n* = 741
	AAS 0	*n* = 30
	Average AAS	55.77
Correlation of AASs and citation numbers		*r* = 0.406**, *p* < 0.01
AAS source	Twitter	24.74
	News	6.40
	Blog	0.61
	Facebook	0.97
	Wiki	0.26
	Google	0.18
Open access		*n* = 262

### Characteristics of the Top-cited and Top-AAS articles

We also conducted a detailed analysis of the citation counts, AASs, study designs, and open access status of the Top-cited and Top-AAS articles (Table 2). Among the Top-cited articles, 92 were cited 90 to 499 times, 5 were cited 500 to 999 times, and 3 were cited ≥1,000 times. Average citation of Top-cited articles was 244.88. There were 57 articles with AASs of 0–79, 34 articles with AASs between 80 and 499, 4 articles with AASs between 500 to 999, and 5 articles with AASs of ≥1,000. Average AAS of Top-cited articles was 225.49. Among the Top-AAS articles, 56 were cited 1 to 89 times, 36 were cited 90 to 499 times, 5 were cited 500 to 999 times, and 3 were cited ≥1,000 times. Average citation of Top-AAS articles was 157.57. There were 83 articles with AASs between 80 and 499, 12 articles with AASs between 500 to 999, and 5 articles with AASs of ≥1,000. Average AAS of Top-AAS articles was 341.6. A weak correlation was found between correlation of AASs and citation numbers in Top-cited articles (*r* = 0.36**, *p* < 0.01) and in Top-AAS articles (*r* = 0.247*, *p* < 0.05). As for the study design, nearly half of Top-cited articles were review (*n* = 47), and a quarter of Top-cited articles were basic study (*n* = 25), whereas nearly half of Top-AAS articles were observational study (*n* = 47), including case control (*n* = 17), case report (*n* = 8), case series (*n* = 6), cohort study (*n* = 13), cross-sectional (*n* = 3). Besides, Top-cited (*n* = 82) and Top-AAS (*n* = 89) articles were both more likely to be open access.

**Table 2:** Characteristics the top-cited and top-AAS articles.

Characteristics		Top-cited	Top-AAS
Citation	Citation ≥1,000	*n* = 3	*n* = 3
	Citation 500–999	*n* = 5	*n* = 5
	Citation 90–499	*n* = 92	*n* = 36
	Citation 1–89	–	*n* = 56
	Average citation	244.88	157.54
AAS	AAS ≥1,000	*n* = 5	*n* = 5
	AAS 500–999	*n* = 4	*n* = 12
	AAS 80–499	*n* = 34	*n* = 83
	AAS 0–79	*n* = 57	–
	Average AAS	225.49	341.6
Correlation of AASs and citation numbers		*r* = 0.36**, *p* < 0.01	*r* = 0.247*, *p* < 0.05
AAS source	Twitter	71.65	118.88
	News	29.43	43.84
	Blog	3.24	3.61
	Facebook	4.18	5.06
	Wiki	1.37	1.06
	Google	0.86	1.03
Observational study	Basic study	*n* = 25	*n* = 25
	Case control	*n* = 11	*n* = 17
	Case report	*n* = 5	*n* = 8
	Case series	*n* = 7	*n* = 6
	Cohort study	*n* = 5	*n* = 13
	Cross-sectional	*n* = 0	*n* = 3
	Review	*n* = 47	*n* = 28
Open access		*n* = 82	*n* = 89

### Research hot topics of CTE

**Figure 3:**
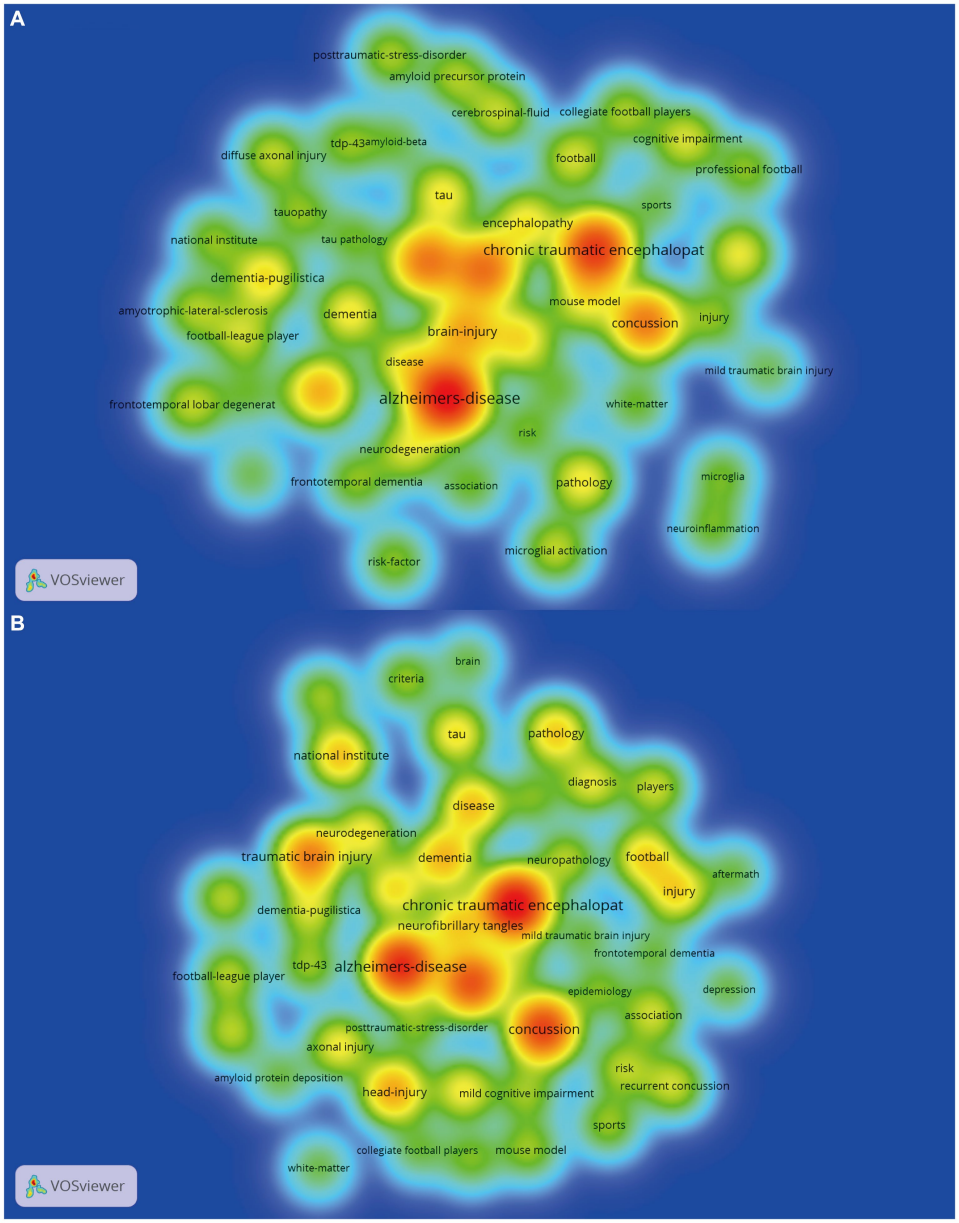
Keywords density visualization map of Top-cited **(A)** vs. Top-AAS **(B)** articles by VOSviewer software. “chronic traumatic encephalopathy,” “Alzheimer’s disease,” “traumatic brain injury,” “concussion” and “brain-injury” were the hot topics both appeared in the Top-cited map and Top-AAS map.

We separately plotted in Figures 3A,B the keyword density visualization map of the Top-cited and Top-AAS articles utilizing VOSviewer, and detailed keywords lists were presented in Table 3. Density views are especially useful for understanding the overall structure of a map and drawing attention to the most important areas on it (29). Each node in the keyword density visualization plot had a color that relied on the density of items at that node. The keywords in the red area appeared more recurrent, indicating the keywords were the most frequently occurring hot topics. We found that “chronic traumatic encephalopathy,” “Alzheimer’s disease,” “traumatic brain injury,” “concussion” and “brain-injury” were the hot topics both appeared in the Top-cited map and Top-AAS map. In addition to the hot keywords that both appeared in Top-cited map and Top-AAS map, we also found that some keywords “amyloid precursor protein,” “cerebrospinal-fluid diffuse,” “axonal injury” and “microglial activation” only appeared in Top-cited map, and “diagnosis,” “criteria” and “neuropathologic assessment” only appeared in Top-AAS map.

**Table 3:** Information of keywords with keywords density visualization map.

	keywords
The common keywords list in Top-cited and Top-AAS articles (According to VOSviewer keyword density visualization map)	chronic traumatic encephalopathy Alzheimer-disease (AD) concussion traumatic brain injury post-traumatic stress disorder brain-injury head-injury dementia disease pathology football neurofibrillary tangles tau encephalopathy neurodegeneration
The unique keywords list in Top-cited articles	diffuse axonal injury microglial activation amyloid precursor protein microglia neuroinflammation risk-factor amyloid-beta
The unique keywords list in Top-AAS articles	diagnosis criteria neuropathologic assessment epidemiology

### Knowledge evolution of CTE

**Figure 4:**
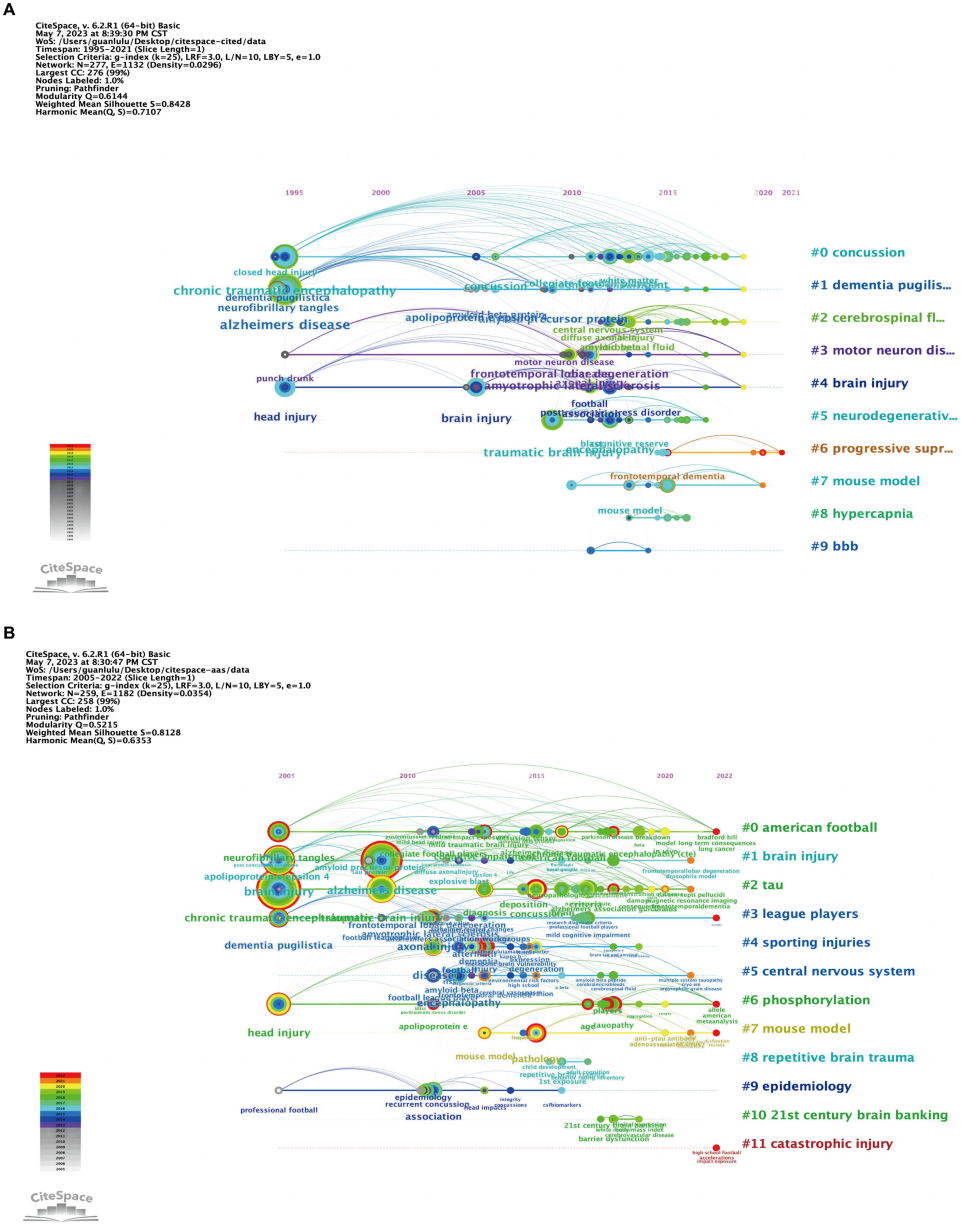
Timeline view of Top-cited **(A)** vs. Top-AAS **(B)** articles. This view represents the appearance of clusters at different time points and time spans. In timeline view of Top-cited **(A)**, there was a total of 9 clusters. In timeline view of Top-AAS **(B)**, there was a total of 11 clusters.

In the CiteSpace timeline view, we could clearly see the evolution of each cluster over time. Each node in the network can be clustered together according to its interconnectivity to form different clusters, with each cluster representing a different professional or disciplinary concept (30). As shown in Figures 4A,B, the cluster and timeline views of the Top-cited and Top-AAS articles were presented, and clusters information were listed in Supplementary Table S2. The active time of each cluster on the timeline reflected the attention given to the research topic in different periods. The modularity value and the mean silhouette score were 0.614 (>0.3), 0.843 (>0.5) in Top-cited and 0.521 (>0.3), 0.813 (>0.5) in Top-AAS, indicating that the structures of clusters were significant and the clusters were efficient and convincing (30). In timeline view of Top-cited, there was a total of 9 clusters. #0 concussion, #1 dementia pugilistica, #3 motor neuron disease and #4 brain injury had been active since 1995, whereas #2 cerebrospinal fluid, #5 neurodegenerative disorders, #8 hypercapnia and #9 bbb began to receive attention in approximately 2010. Only #6 progressive supranuclear palsy and #7 mouse model still maintained a high degree of activity until now. In timeline view of Top-AAS, there was a total of 11 clusters. #0 American football, #1 brain injury, #2 tau, #3 league players, #6 phosphorylation and #9 epidemiology had been active since 2005, and #4 brain injury, #5 central nervous system, #7 mouse model, #8 repetitive brain trauma and #10 21st century brain bank had received attention since 2012. Notably, #0 American football, #3 league players, #6 phosphorylation, #7 mouse model and #11 catastrophic injury were relatively frontier research direction, which has received widespread attention today.

### Overlaps between Top-cited and Top-AAS articles

There were 44 articles that overlapped between Top-cited and Top-AAS articles, indicating the 44 articles both had high citations and high AASs. We called them High-High articles in the following discussion and more details were available in the Supplementary Table S3. From Table 4, There were 36 articles were cited 90 to 499 times, 5 were cited 500 to 999 times, and 3 were cited ≥1,000 times. Among the High-High articles, 35 articles with AASs between 79 and 499, 4 articles with AASs between 500 to 999, and 5 articles with AASs of ≥1,000. It was evident that the average citation count and the average AAS of these High-High articles were significantly high, averaging at 317.89 and 476.2, respectively. There was a moderate correlation (*r* = 0.415**, *p* < 0.01) between citation numbers and AASs in the High-High articles. Most of the High-High articles were open access (*n* = 40). A considerable proportion of the High-High articles had more than 4 collaborating institutions (*n* = 36) and more than 5 authors (*n* = 33). Over half of the High-High articles (*n* = 25) published in journals with impact factor greater than 10. As for study design, the most common type of the High-High articles was observational study (*n* = 20), followed by review (*n* = 12). As for the characteristics of title, there were over half of High-High articles (*n* = 27) with number of words in title shorter than 12, nearly 1/3 articles (*n* = 12) with titles separated by a colon or ended with a question mark, and nearly 1/3 articles (*n* = 12) had declarative titles that used a verb (usually past tense or present tense).

**Table 4:** Characteristics the high-high articles.

Characteristics		Results
Citation	Citation ≥1,000	*n* = 3
	Citation 500–999	*n* = 5
	Citation 90–499	*n* = 36
	Average citation	317.89
AAS	AAS ≥1,000	*n* = 5
	AAS 500–1,000	*n* = 4
	AAS 79–499	*n* = 35
	Average AAS	476.2
Correlation of AASs and citation numbers		*r* = 0.415**, *p* < 0.01
AAS source	Twitter	78.56
	News	29.89
	Blog	2.89
	Facebook	2.78
	Wiki	1.56
	Google	2.56
Open access		*n* = 40
Affiliations ≥4		*n* = 36
Authors ≥5		*n* = 33
Journal IF >10		*n* = 25
Study design	Observational study	*n* = 20
	Review	*n* = 15
Number of words in titles ≤12		*n* = 27
Presence of a colon or question in title		*n* = 12
Declarative title (used a verb)		*n* = 12

## Discussion

Over the last few decades, there has been an amount of breakthrough about CTE research, including biomarkers for differential diagnosis (12), *in-vivo* positron emission tomography (PET) tau imaging (11), neuropathological diagnosis criteria (8), and tau filaments structures determination (13). These developments were evidenced by the vast number of articles published on CTE. Although there were some bibliometric or altmetric studies on CTE in the literature (25–27), this study is the first of its kind to combine bibliometric and altmetric analysis that may provide a more comprehensive understanding of the CTE field. In this study, we identified the characteristics of total CTE articles and examined the traits and commonalities/differences between Top-cited and Top-AAS articles. We also found there were 44 articles that overlapped between Top-cited and Top-AAS articles and reported the characteristics of them. This study revealed the shared and distinct research interests in CTE among academia and the general public, and could provide researchers with suggestions and inspirations that might enhance the impact of their research work about CTE in the future.

### Topic of interest in the scientific community and the general public

Through Top-cited and Top-AAS articles, we could see that there was considerable overlap between the topics covered by the two representative lists. From the common keywords list that appeared in Top-cited and Top-AAS articles (Table 3), we could observe that both the general public and the scientific community were particularly interested in the risk factors for neurodegenerative diseases such as CTE, AD, and dementia, especially the role of injury and concussion in their development. It is important to note that not all head injuries lead to CTE, and the exact relationship between head trauma and CTE is still not fully understood (31). The link between injuries, concussions, and CTE has become a major area of research in recent years, particularly in the context of professional sports. Furthermore, numerous studies indicated a correlation between traumatic brain injury (TBI) and post-traumatic stress disorder (PTSD), and demonstrated role of TBI in rendering individuals more susceptible to developing PTSD (32, 33). Moreover, we also found that the pathology of CTE captured the attention of the general public and scientific community. The accumulation of abnormal tau protein in the brain into neurofibrillary tangles is one of hallmarks of CTE (34). In addition to CTE, abnormal aggregation of tau protein also occurs in other neurodegenerative diseases like AD and dementia (32, 35). They may differ in the location, morphology, and severity of aggregation (12). More research is still needed to explore the specific mechanisms and impacts of tau aggregation in these neurodegenerative diseases. Besides, we could see from Figure 4 that mouse model is the research frontiers in the field of CTE and garnered both public and scientific attention. Establishing CTE-like disease in models (non-impact head acceleration, blast wave, weight drop, fluid percussion, and controlled cortical impact models) could provide a better understanding of neurobiological molecular pathways (36).

Substantial differences were also found between the characteristics of Top-cited and Top-AAS articles. By analyzing the unique keywords list in Top-cited articles, we could know that scientists were interested in studying the causes and characteristics of neurodegenerative diseases. Diffuse axonal injury, microglial activation, accumulation of amyloid precursor protein, neuroinflammation, and formation of beta-amyloid plaques are key factors in the development and progression of CTE. These factors may interact with each other, leading to dysfunction and death of brain cells (37). Research into these pathological features of CTE is ongoing, and investigating the underlying mechanisms may provide important insights into potential therapeutic targets for the disease.

However, from the unique keywords list in Top-AAS articles, we could know the general public has become increasingly concerned about the detection and prevention of CTE. Diagnosing CTE requires examining brain tissue under a microscope to detect the presence of specific neuropathological features, and there is currently no reliable method for diagnosing CTE in a living person (8). However, identifying ante-mortem prognosis of CTE instead of post-mortem diagnosis is of significant societal importance. PET imaging is being studied as a potential tool for detecting early signs of the disease in living individuals by detecting the concentration of tracer injected into the body (11). Recently, Boston University CTE Center examined the association between antemortem tracer flortaucipir-PET uptake and postmortem p-tau neuropathology in six deceased former elite American football players. Findings from this PET-to-autopsy case series suggested that PET tracer flortaucipir may be useful for detecting high stage CTE neuropathology (38). In future, more research is needed to refine this technique and develop it into a reliable diagnostic tool for CTE.

### The shared characteristics of High-High articles

There were 44 articles that overlapped between the Top-cited and Top-AAS articles. The 44 High-High articles displayed both high number of citations and high AASs, indicating that these articles had both significant scholarly impact and great public attention. It can be concluded that certain topics or findings in CTE field have captured the attention of both the scientific community and the general public. This phenomenon did not appear in other similar studies (23, 24, 39). By examining the shared characteristics of High-High articles, we identified certain traits that may potentially contribute to their high citation rates and high AASs.

Journal impact: After conducting our analysis, we found that a significant portion of the High-High articles were featured in journals with notably high impact factors, such as *Nature, Science, JAMA, Lancet, New England Journal of Medicine*, and *British Journal of Sports Medicine*. This observation implied that publishing in a journal with a higher impact factor may be a viable strategy for achieving High-High outcomes, as is the case in other fields (40, 41).

Scientific collaboration: In 44 High-High articles, most article possessed more than five authors and involved three or more research institutions. Our analysis revealed a compelling link between research collaboration (individual or institutional) and the attainment of High-High outcomes. A number of studies found that multi-authorship increases above all the probability to be cited by others (42), and papers published by the cooperation of authors from several organizations gather significantly more citations than papers authored by authors from one organization (43, 44). Besides, it is maintained that papers with international collaboration have a greater impact than papers with national collaborations because of their greater quality and prestige (45). However, in High-High articles, institutional cooperation was still limited to Boston University and Harvard University, and remained primarily domestic cooperation within the United States. The strong international collaborations with other nations or regions have not found and they have not formed such a dense collaboration network. As noted in a previous bibliometric analysis of CTE, it was imperative that institutions from around the globe were motivated to engage in the discourse surrounding sports-related CTE and provide their diverse viewpoints (26). This will enable the establishment of a more comprehensive and diverse international network of collaborations, beyond the United States, to further advance the understanding and knowledge of CTE.

Study design: Our analysis revealed that observational studies were the most prevalent in High-High articles. Most of the observational studies were on diagnoses features (neuropathological and clinical) of CTE. However, typically, review papers tend to garner more impacts than research papers (43). AAS reflects the impact of disseminating research based on the interests of the general public, exhibiting greater interest in common clinical medicine topics than in complex, fundamental issues. The study subjects involved in these observational studies were the core population in the field of CTE that attracted public attention and spark discussion. These observational studies acted as building blocks of research and predominated among classics. The article titled “Clinicopathological Evaluation of Chronic Traumatic Encephalopathy in Players of American Football” published in *JAMA* July 25, 2017 with extremely high score 4,770 reported that 110 of 111 (99%) former football players were suspected of having CTE (19). Another article titled “The spectrum of disease in chronic traumatic encephalopathy” analyzed post-mortem brains obtained from a cohort of 85 subjects with histories of repetitive mild traumatic brain injury and found evidence of CTE in 68 subjects (46). These observational studies with stunning results and conclusions have sent shockwaves through the scientific environment and the general public.

Characteristics of the title: Through analyzing the title of High-High articles, we found that many article titles have certain characteristics, including brevity (Number of words in titles <12), attention-grabbing (Presence of a colon or question in title), and accuracy (Declarative: used a verb). An attractive, simple, understandable, concise, and informative title for articles might be more attractive to readers (47). This phenomenon in other disciplines also applied to our research, which the titles of High-High articles were more readable than titles that use complex terminologies. There was evidence that articles with shorter titles were more likely to have advantages of citations (40, 48, 49). and articles with declarative/interrogative titles were associated with higher AASs (40, 50). However, due to the limited scope of our research, more specialized studies of association between characteristics of tile and article impact, especially with higher sample size, are required to generalize the findings of our study.

Accessibility of papers: In our study, most of High-High articles were open access. Papers published in open access journals were cited more often in comparison to papers published in non-open access journals (51, 52). The impact of open access is not only reflected in citation, the potential for more extensive research dissemination inherent in the open access option may translate into greater reach and social media attention (53). When an article is more accessible, it has the potential to reach more readers and be cited more frequently, ultimately increasing its visibility and impact within its field.

Sharing activities on social media: We have tracked the AAS sources of the High-High articles, and found that news was a great contributor to the overall score. Our study provided objective evidence that these topics received a great deal of attention in prominent news media outlets such as CNN news, Yahoo, and TIMES. Besides, Twitter activity was one of the major contributors to the final AAS of almost all articles, which was consistent with other studies (54–56). We discovered that in addition to comments from the general public, many tweets about an article also came from official Twitter accounts of its published journal (@ScienceMagazine, @NaturePortfolio, @NEJM @JAMA_current and @Brain1878), research institution (@ShorterLabGrou, @UCSFmac, @Carter_Lab, @MADLab1, and @bu_cte), author team, and other relevant journals. In the era of social media today, various scholar organizations and individuals can use platforms such as Twitter/Blog to share their views and opinions, and promote their research results and perspectives. Moreover, many journals themselves have their own official Twitter accounts, particularly top journals such as *Lancet, JAMA, Nature*, and *Science*, and tweet their recent articles regularly. Many studies from other disciplines have confirmed the effectiveness of Twitter in increasing the impact of articles (57–60). The role of social platforms in the dissemination of research is being increasingly recognized.

**Figure 5:**
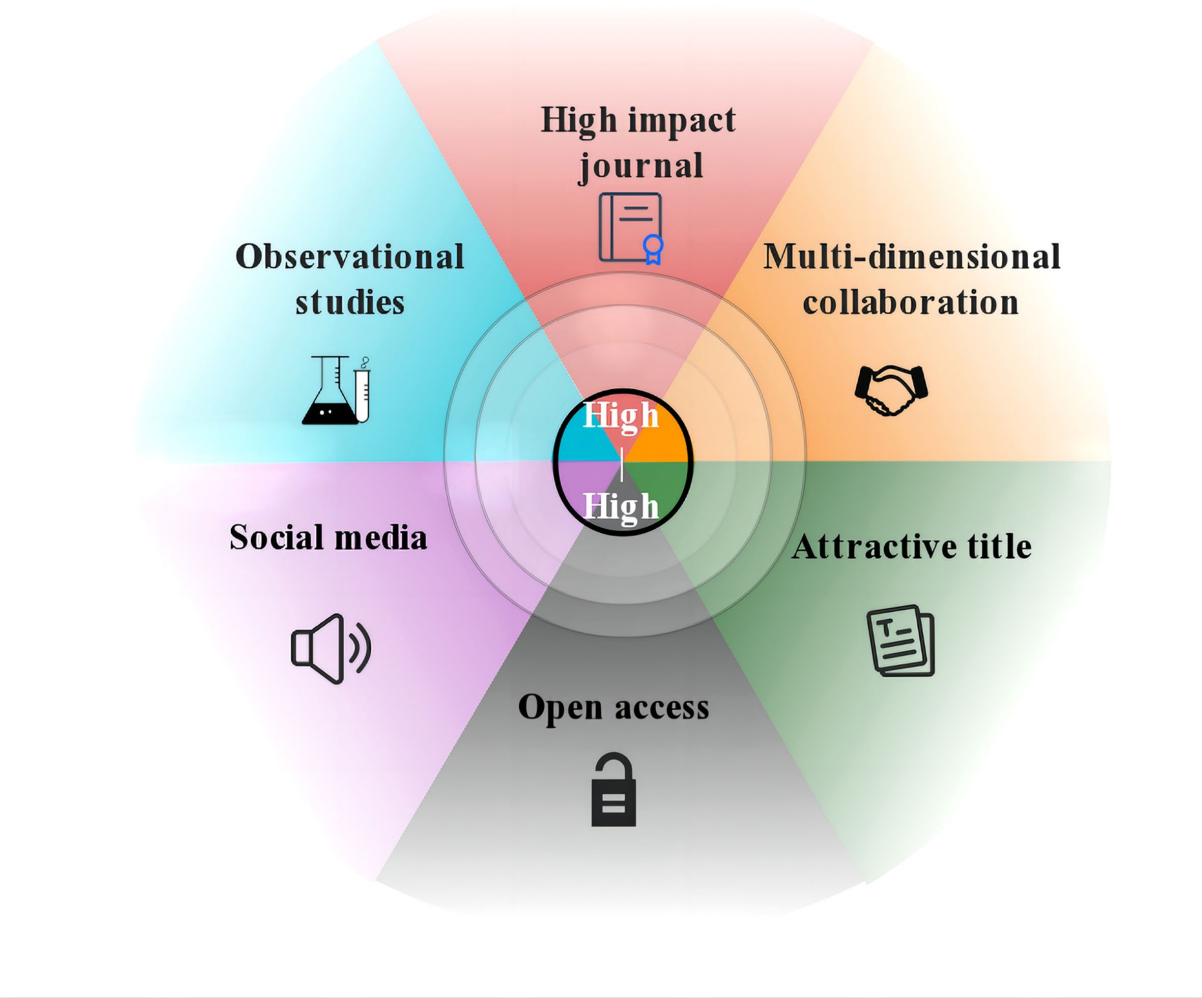
The shared characteristics of High-High articles.

In summary, through analyzing the High-High articles of Top-cited and Top-AAS, we summarized the commonalities, including: publishing in high-impact journals; having multilateral collaborations; observational studies; having attractive titles; open access; and using social media tools (Figure 5). It might provide valuable insights for researchers to improve the quality and impact of their future studies in CTE field.

### Associations between number of citations and AASs

Unlike findings reported by other similar studies(61–63), our results showed significant positive correlations between AAS and citation with varied strength for all 788 articles, Top-cited, Top-AAS, and High-High datasets. This difference between our work and other study may attribute to the differential research field. Although such associations were observed in our study, there was uncertainty surrounding the ability of the AAS score to accurately reflect traditional bibliometrics. Moreover, it is crucial to emphasize that online attention can be positive or negative. A controversial article may garner public interest, thereby increase the AAS of the article, while the impact of the article on the scientific community might not align with the level of public attention. Thus, AAS should be used as a complementary measure to aid researchers in appraising social media presence and evaluate the quality and impact of an article along with traditional metrics.

In the last, as previous study mentioned, several journals including *the American Journal of Epidemiology*, have appointed associate editors of social media to enhance conversations about the work they publish on social media (64). We would recommend that journals and researchers use various methods of social media promotion. Meanwhile, there is a need for increased incorporation of experimental study designs to better determine the effectiveness of different communication strategies across various channels. Twitter, News, Facebook, infographics, blog posts, virtual abstracts, and podcasts, each of these modalities has its unique characteristics and audience, and may have different effects on dissemination and readership of research findings. Today, the impact of a scientific work extends far beyond traditional citation metrics. An article might be read in order to create its value in clinical decision-making, academic educational content, educational content for the general public, development of a policy document or a guideline, public discourse and awareness, etc. Confronted with the crucial issue of CTE in people’s daily lives, CTE researchers should pay more attention to social media to introduce scientific achievements in simple and easy-to-understand language, and convey the value and significance of science to the public. Furthermore, researchers and policymakers could connect the public and academics to improve public understanding of CTE related knowledge, and share clinical guides to enhance public health.

## Conclusion

This study presented bibliometric and altmetric overviews of CTE-linked literature and compared the most discussed articles on CTE, highlighting the similarities and differences in the understanding of CTE between the scientific community and the general public. There was a moderate correlation between AASs and citation numbers of the total articles we gathered. Both the general public and the scientific community were particularly interested in the risk factors and pathology of CTE, and scientists were interested in studying the causes and characteristics of neurodegenerative diseases while the public has become increasingly concerned about the detection and prevention of CTE. We determined that there was considerable overlap between articles that scientists and the general public most concerned, and analyzed the potential shared characteristics of the overlapped articles. This study, which combines altmetric and traditional metrics, may provide a more comprehensive description of scientific research output, offering a point of reference for future CTE research in the selection of study topics and directions.

### Limitations

A possible limitation of this type of study was that a single database may lead to some missed publications and limit the scope of the analyses and results. It might be possible to find different insights if a larger sample of articles was included. In further studies, more various databases, such as Google Scholars, will be considered to collect data. Another limitation was the time dependence of both citation and social media mentions. The number of citations tends to increase with time, and citations rarely decrease. Unlike citations, the AAS is dynamic and can rise or fall. Lastly, given altmetric data’s recency bias, emerging research is more likely to be tweeted because Twitter was not available as a platform before 2007.

## Data availability statement

The original contributions presented in the study are included in the article/Supplementary material, further inquiries can be directed to the corresponding authors.

## Author contributions

LG: Conceptualization, Data curation, Formal analysis, Investigation, Methodology, Software, Visualization, Writing – original draft. JT: Data curation, Formal analysis, Methodology, Writing – review & editing. BQ: Data curation, Formal analysis, Software, Visualization, Writing – review & editing. YC: Formal analysis, Software, Visualization, Writing – review & editing. ET: Data curation, Formal analysis, Writing – review & editing. JP: Conceptualization, Investigation, Supervision, Writing – review & editing. YZ: Conceptualization, Investigation, Methodology, Supervision, Writing – review & editing.

## Footnotes

^1^ https://www.altmetric.com/
