# Supplementary material for: The bibliometric and altmetric analysis of chronic traumatic encephalopathy research: how great is the impact?
Source: Front Neurol. 2024 Feb 8;15:1294125. doi: 10.3389/fneur.2024.1294125 (PMC10883053; doi:10.3389/fneur.2024.1294125)
Supplement: Supplementary file 1 [file Table_1.DOCX]

**Supplementary Material**

**Table S1. Descriptions and relative weight of** **each online source toward the AAS of an article∗.**

| Online source | Description | Weight |
| --- | --- | --- |
| News | Mainstream magazines, newspaper, and TV channels (e.g., The New Yorker, USA Today, CNN, BuzzFeed) | 8 |
| Blogs | Web 2.0 based Web sites that use user-generated content (e.g., Tumblr and WordPress) | 5 |
| Wikipedia | Microblogging social network platform | 3 |
| Twitter | Microblogging social network platform | 1 |
| Google+ | Social networking service that uses text and media communications | 1 |
| Weibo | Chinese social media platform for microblogging | 1 |
| Facebook | Social networking service that uses text and media communications | 0.25 |

∗Source of relative weight: <https://www.altmetric.com/>.

**Table S2**. **Clusters list of timeline views of the Top-cited and Top-AAS articles.**

| Top-cited | Top-AAS |
| --- | --- |
| #0 concussion  #1 dementia pugilistica  #2 cerebrospinal fluid  #3 motor neuron disease  #4 brain injury  #5 neurodegenerative disorders  #6 progressive supranuclear palsy  #7 mouse model  #8 hypercapnia  #9 bbb | #0 american football  #1 brain injury  #2 tau  #3 league players  #4 brain injury  #5 central nervous system  #6 phosphorylation  #7 mouse model  #8 repetitive brain trauma  #9 epidemiology  #10 21st century brain bank  #11 catastrophic injury |

**Table S3**. **High-High articles overlapped between Top-cited and Top-AAS articles.**

| Title | Citation | AAS | Author | Affiliation | Journal | Study design | Open access status | News | Blog | Twitter |
| --- | --- | --- | --- | --- | --- | --- | --- | --- | --- | --- |
| Chronic traumatic encephalopathy in athletes: Progressive tauopathy after repetitive head injury | 1493 | 1012 | Mckee, AC; Cantu, RC; Nowinski, CJ; Hedley-Whyte, ET; Gavett, BE; Budson, AE; Santini, VE; Lee, HS; Kubilus, CA; Stern, RA | Geriatric Research Education & Clinical Center; Boston University; Harvard University; Harvard Medical School; Massachusetts General Hospital | *Journal of Neuropathology and Experimental Neurology* (3.84) | Review | True | 147 | 19 | 71 |
| The spectrum of disease in chronic traumatic encephalopathy | 1281 | 2481 | McKee, AC; Stein, TD; Nowinski, CJ; Stern, RA; Daneshvar, DH; Alvarez, VE; Lee, HS; Hall, G; Wojtowicz, SM; Baugh, CM; Riley, DO; Kubilus, CA; Cormier, KA; Jacobs, MA; Martin, BR; Abraham, CR; Ikezu, T; Reichard, RR; Wolozin, BL; Budson, AE; Goldstein, LE; Kowall, NW; Cantu, RC | US Department of Veterans Affairs; Veterans Health Administration (VHA); Harvard University; VA Boston Healthcare System; Boston University; University of Massachusetts System; University of Massachusetts Lowell; University; Mayo Clinic; | *Brain* (16.173) | Case control | True | 422 | 29 | 223 |
| Blood-brain barrier breakdown in Alzheimer disease and other neurodegenerative disorders | 1137 | 158 | Sweeney, MD; Sagare, AP; Zlokovic, BV | University of Southern California; University of Southern California | *Nature Reviews Neurology* (42.347) | Review | True | 3 | 1 | 241 |
| Microglia in neurodegeneration | 661 | 540 | Hickman, S;  Izzy, S; Sen, P; Morsett, L;  El Khoury, J | Harvard University; Massachusetts General Hospital; Harvard Medical School | *Nature Neuroscience* (29.887) | Review | True | 66 | 5 | 148 |
| Chronic traumatic encephalopathy in a National Football League player | 654 | 774 | Omalu, BI; DeKosky, ST; Minster, RL; Kamboh, MI; Hamilton, RL; Wecht, CH | Pennsylvania Commonwealth System of Higher Education (PCSHE); University of Pittsburgh; | *Neurosurgery* (5.716) | Case report | False | 92 | 20 | 125 |
| Chronic traumatic encephalopathy in blast-exposed military veterans and a blast neurotrauma mouse model | 642 | 359 | Goldstein, LE; Fisher, AM; Tagge, CA; Zhang, XL; Velisek, L; Sullivan, JA; Upreti, C; Kracht, JM; Ericsson, M; Wojnarowicz, MW; Goletiani, CJ; Maglakelidze, GM; Casey, N; Moncaster, JA; Minaeva, O; Moir, RD; Nowinski, CJ; Stern, RA; Cantu, RC; Geiling, J; Blusztajn, JK; Wolozin, BL; Ikezu, T; Stein, TD; Budson, AE; Kowall, NW; Chargin, D; Sharon, A; Saman, S; Hall, GF; Moss, WC; Cleveland, RO; Tanzi, RE; Stanton, PK; McKee, AC | Boston University; New York Medical College; Harvard Medical School; Harvard University; Massachusetts General Hospital; US Department of Veterans Affairs; VA Boston Healthcare System; University of Massachusetts System; University of Massachusetts Lowell; University of Oxford; United States Department of Energy (DOE); Lawrence Livermore National Laboratory | *Science Translational Medicine* (22.173) | Case  Series/Basic study | True | 41 | 5 | 42 |
| Clinicopathological evaluation of chronic traumatic encephalopathy in players of American football | 547 | 4770 | Mez, J; Daneshvar, DH; Kiernan, PT; Abdolmohammadi, B; Alvarez, VE; Huber, BR; Alosco, ML; Solomon, TM; Nowinski, CJ; McHale, L; Cormier, KA; Kubilus, CA; Martin, BM; Murphy, L; Baugh, CM; Montenigro, PH; Chaisson, CE; Tripodis, Y; Kowall, N; Weuve, J; McClean, MD; Cantu, RC; Goldstein, LE; Katz, DI; Stern, RA; Stein, TD; McKee, AC | Boston University; Boston University; Stanford University; US Department of Veterans Affairs; Veterans Health Administration (VHA); Harvard University; VA Boston Healthcare System; Boston Children's Hospital; | *Journal of the American Medical Association* (101.129) | Case series | True | 838 | 65 | 1736 |
| The first NINDS/NIBIB consensus meeting to define neuropathological criteria for the diagnosis of chronic traumatic encephalopathy | 516 | 290 | McKee, AC; Cairns, NJ; Dickson, DW; Folkerth, RD; Keene, CD; Litvan, I; Perl, DP; Stein, TD; Vonsattel, JP; Stewart, W; Tripodis, Y; Crary, JF; Bieniek, KF; Dams-O'Connor, K; Alvarez, VE; Gordon, WA | Boston University; US Department of Veterans Affairs; Veterans Health Administration (VHA); Harvard University; VA Boston Healthcare System; Washington University (WUSTL); Mayo Clinic; Harvard University; Brigham & Women's Hospital; Harvard Medical School; University of Washington Seattle; University of California System; University of California San Diego; Uniformed Services University of the Health Sciences - USA; Columbia University; University of Glasgow; Queen Elizabeth University Hospital (QEUH); Icahn School of Medicine at Mount Sinai; Icahn School of Medicine at Mount Sinai | *Acta Neuropathologica*  (21.534) | Case report | True | 40 | 4 | 107 |
| Chronic traumatic encephalopathy: A potential late effect of sport-related concussive and subconcussive head trauma | 450 | 330 | Gavett, BE; Stern, RA; McKee, AC | Boston University; | *Clinics in Sports Medicine* (2.668) | Case series | True | 40 | 7 | 35 |
| Epidemiology of mild traumatic brain injury and neurodegenerative disease | 344 | 84 | Gardner, RC; Yaffe, K | University of California System; University of California San Francisco; US Department of Veterans Affairs; Veterans Health Administration (VHA); San Francisco VA Medical Center; | *Molecular and Cellular Neuroscience* (4.016) | Review | True | 6 | 4 | 14 |
| Novel tau filament fold in chronic traumatic encephalopathy encloses hydrophobic molecules | 336 | 448 | Falcon, B; Zivanov, J; Zhang, WJ; Murzin, AG; Garringer, HJ; Vidal, R; Crowther, RA; Newell, KL; Ghetti, B; Goedert, M; Scheres, SHW | MRC Laboratory Molecular Biology; Indiana University System; Indiana University Bloomington; University of Kansas; University of Kansas Medical Center | *Nature* (63.581) | Basic study | True | 30 | 5 | 538 |
| Clinical presentation of chronic traumatic encephalopathy | 324 | 149 | Stern, RA; Daneshvar, DH; Baugh, CM; Seichepine, DR; Montenigro, PH; Riley, DO; Fritts, NG; Stamm, JM; Robbins, CA; McHale, L; Simkin, I; Stein, TD; Alvarez, VE; Goldstein, LE; Budson, AE; Kowall, NW; Nowinski, CJ; Cantu, RC; McKee, AC | Boston University; Department of Veterans Affairs; Veterans Health Administration (VHA); Harvard University; VA Boston Healthcare System; | *Neurology* (11.786) | Case series | True | 16 | 1 | 25 |
| Role of subconcussion in repetitive mild traumatic brain injury: A review | 313 | 378 | Bailes, JE; Petraglia, AL; Omalu, BI; Nauman, E; Talavage, T | NorthShore University Health System; University of Chicago; University of Rochester; University of California System; University of California Davis; Purdue University System; Purdue University; Purdue University West Lafayette Campus; | *Journal of Neurosurgery* (5.266) | Review | False | 40 | 3 | 64 |
| A systematic review of potential long-term effects of sport-related concussion | 312 | 212 | Manley, G; Gardner, AJ; Schneider, KJ; Guskiewicz, KM; Bailes, J; Cantu, RC; Castellani, RJ; Turner, M; Jordan, BD; Randolph, C; Dvorak, J; Hayden, KA; Tator, CH; McCrory, P; Iverson, GL | University of California System; University of California San Francisco; University of Newcastle; John Hunter Hospital; University of Calgary; Alberta Childrens Hospital; University of Calgary; University of North Carolina; NorthShore University Health System; University of Chicago; Boston University; Western Michigan University; Loyola Schulthess Clinic; University of Zurich; University of Calgary; Krembil Research Institute; University of Toronto; University Health Network Toronto; Florey Institute of Neuroscience & Mental Health; Harvard University; Harvard Medical School; Harvard University; Spaulding Rehabilitation Hospital; Harvard University; Massachusetts General Hospital | *British Journal of Sports Medicine* (18.705) | Review | True | 17 | 0 | 160 |
| Chronic traumatic encephalopathy: neurodegeneration following repetitive concussive and subconcussive brain trauma | 306 | 84 | Baugh, CM; Stamm, JM; Riley, DO; Gavett, BE; Shenton, ME; Lin, A; Nowinski, CJ; Cantu, RC; McKee, AC; Stern, RA | Boston University; University of Colorado System; University of Colorado at Colorado Springs; Harvard University; Brigham & Women's Hospital; Harvard Medical School; Brigham & Women's Hospital; | *Brain Imaging and Behavior* (3.807) | Review | False | 9 | 3 | 3 |
| The neuropathology of chronic traumatic encephalopathy | 302 | 216 | McKee, AC;  Stein, TD; Kiernan, PT; Alvarez, VE | US Department of Veterans Affairs; Veterans Health Administration (VHA); Harvard University; VA Boston Healthcare System; Boston University; | *Brain Pathology* (7.463) | Basic study | True | 26 | 3 | 15 |
| Cumulative head impact exposure predicts later-life depression, apathy, executive dysfunction, and cognitive impairment in former high school and college football players | 284 | 252 | Montenigro, PH; Alosco, ML; Martin, BM; Daneshvar, DH; Mez, J; Chaisson, CE; Nowinski, CJ; Au, R; Mckee, AC; Cantu, RC; McClean, MD; Stern, RA; Tripodis, Y | Boston University; Framingham Heart Study; Boston University; US Department of Veterans Affairs; Veterans Health Administration (VHA); Harvard University; VA Boston Healthcare System | *Journal of Neurotrauma* (5.233) | Cohort study | True | 19 | 0 | 145 |
| Antibody against early driver of neurodegeneration cis P-tau blocks brain injury and tauopathy | 284 | 210 | Kondo, A; Shahpasand, K; Mannix, R; Qiu, JH; Moncaster, J; Chen, CH; Yao, YD; Lin, YM; Driver, JA; Sun, Y; Wei, S; Luo, ML; Albayram, O; Huang, PY; Rotenberg, A; Ryo, A; Goldstein, LE; Pascual-Leone, A; McKee, AC; Meehan, W; Zhou, XZ; Lu, KP | Harvard University; Beth Israel Deaconess Medical Center; Harvard Medical School; Harvard University; Beth Israel Deaconess Medical Center; Harvard Medical School; Boston Children's Hospital; Boston University; Geriatric Research Education & Clinical Center;  US Department of Veterans Affairs; Veterans Health Administration (VHA); VA Boston Healthcare System; Yokohama City University; Beth Israel Deaconess Medical Center; | *Nature* (63.581) | Basic study | True | 14 | 8 | 75 |
| The chronic and evolving neurological consequences of traumatic brain injury | 242 | 159 | Wilson, L; Stewart, W; Dams-O'Connor, K; Diaz-Arrastia, R; Horton, L; Menon, DK; Polinder, S | University of Stirling; Queen Elizabeth University Hospital (QEUH); University of Glasgow; Icahn School of Medicine at Mount Sinai; University of Pennsylvania; Pennsylvania Medicine; Cambridge University Hospitals NHS Foundation Trust; Addenbrooke's Hospital; University of Cambridge;  Erasmus University Rotterdam; Erasmus MC | *Lancet Neurology* (50.844) | Basic study | True | 9 | 0 | 193 |
| Concussion, microvascular injury, and early tauopathy in young athletes after impact head injury and an impact concussion mouse model | 238 | 1645 | Tagge, CA; Fisher, AM; Minaeva, OV; Gaudreau-Balderrama, A; Moncaster, JA; Zhang, XL; Wojnarowicz, MW; Casey, N; Lu, HY; Kokiko-Cochran, ON; Saman, S; Ericsson, M; Onos, KD; Veksler, R; Senatorov, VV; Kondo, A; Zhou, XZ; Miry, O; Vose, LR; Gopaul, KR; Upreti, C; Nowinski, CJ; Cantu, RC; Alvarez, VE; Hildebrandt, AM; Franz, ES; Konrad, J; Hamilton, JA; Hua, N; Tripodis, Y; Anderson, AT; Howell, GR; Kaufer, D; Hall, GF; Lu, KP; Ransohoff, RM; Cleveland, RO; Kowall, NW; Stein, TD; Lamb, BT; Huber, BR; Moss, WC; Friedman, A; Stanton, PK; McKee, AC; Goldstein, LE | Boston University; University; New York Medical College; Boston University; Cleveland Clinic Foundation; University of Massachusetts System; University of Massachusetts Lowell; Harvard University; Harvard Medical School; Jackson Laboratory;  Ben Gurion University;  University of California System; University of California Berkeley; Beth Israel Deaconess Medical Center;  US Department of Veterans Affairs; Veterans Health Administration (VHA); Harvard University; VA Boston Healthcare System; United States Department of Energy (DOE); Lawrence Livermore National Laboratory;  University of Oxford; US  Dalhousie University; University System of Ohio;  Ohio State University; Biogen; Indiana University System; Indiana University Bloomington | *Brain* (16.173) | Basic study | True | 251 | 18 | 282 |
| Military-related traumatic brain injury and neurodegeneration | 228 | 413 | McKee, AC; Robinson, ME | US Department of Veterans Affairs; Veterans Health Administration (VHA); Harvard University;  VA Boston Healthcare System; Boston University; | *Alzheimers & Dementia* (20.298) | Case report | True | 21 | 2 | 323 |
| Chronic traumatic encephalopathy pathology in a neurodegenerative disorders brain bank | 186 | 323 | Bieniek, KF; Ross, OA; Cormier, KA; Walton, RL; Soto-Ortolaza, A; Johnston, AE; DeSaro, P; Boylan, KB; Graff-Radford, NR; Wszolek, ZK; Rademakers, R; Boeve, BF; McKee, AC; Dickson, DW | Mayo Clinic;  US Department of Veterans Affairs; Veterans Health Administration (VHA); Harvard University; VA Boston Healthcare System; Boston University; | *Acta Neuropathologica* (21.534) | Case control | True | 43 | 5 | 68 |
| Persistent, long-term cerebral white matter changes after sports-related repetitive head impacts | 185 | 92 | Bazarian, JJ; Zhu, T; Zhong, JH; Janigro, D; Rozen, E; Roberts, A; Javien, H; Merchant-Borna, K; Abar, B; Blackman, EG | University of Rochester; Cleveland Clinic Foundation; Hamilton College | *Plos One* (4.069) | Cohort study | True | 5 | 5 | 28 |
| LRP1 is a master regulator of tau uptake and spread | 183 | 230 | Rauch, JN; Luna, G; Guzman, E; Audouard, M; Challis, C; Sibih, YE; Leshuk, C; Hernandez, I; Wegmann, S; Hyman, BT; Gradinaru, V; Kampmann, M; Kosik, KS | University of California System; University of California Santa Barbara; California Institute of Technology; Helmholtz Association;  German Center for Neurodegenerative Diseases (DZNE); Harvard University; Harvard Medical School; Massachusetts General Hospital; | *Nature* (63.581) | Basic study | True | 16 | 2 | 233 |
| Long-term consequences: Effects on normal development profile after concussion | 172 | 128 | Daneshvar, DH; Riley, DO; Nowinski, CJ; McKee, AC; Stern, RA; Cantu, RC | Boston University; Harvard University; Brigham & Women's Hospital; Boston University | *Physical Medicine and Rehabilitation Clinics of North America* (3.315) | Review | True | 14 | 1 | 12 |
| Cumulative head impact burden in high school football | 163 | 342 | Broglio, SP; Eckner, JT; Martini, D; Sosnoff, JJ; Kutcher, JS; Randolph, C | University of Michigan System; University of Michigan; Loyola University Chicago | *Journal of Neurotrauma* (5.233) | Cohort study | True | 55 | 5 | 3 |
| Chronic traumatic encephalopathy in an Iraqi war veteran with posttraumatic stress disorder who committed suicide | 161 | 79 | Omalu, B; Hammers, JL; Bailes, J; Hamilton, RL; Kamboh, MI; Webster, G; Fitzsimmons, RP | University of California System; University of California Davis; West Virginia University; Pennsylvania Commonwealth System of Higher Education (PCSHE); University of Pittsburgh; | *Neurosurgical Focus* (4.994) | Case report | False | 4 | 3 | 17 |
| Dementia resulting from traumatic brain injury: What is the pathology? | 160 | 133 | Shively, S;  Scher, AI; Perl, DP; Diaz-Arrastia, R | Uniformed Services University of the Health Sciences - USA; Henry M. Jackson Foundation for the Advancement of Military Medicine, Inc; | *Archives of Neurology* (7.249) | Review | True | 9 | 7 | 23 |
| Microglial neuroinflammation contributes to tau accumulation in chronic traumatic encephalopathy | 153 | 211 | Cherry, JD; Tripodis, Y; Alvarez, VE; Huber, B; Kiernan, PT; Daneshvar, DH; Mez, J; Montenigro, PH; Solomon, TM; Alosco, ML; Stern, RA; Mckee, AC; Stein, TD | Boston University; US Department of Veterans Affairs; Veterans Health Administration (VHA); Harvard University; VA Boston Healthcare System | *Acta Neuropathologica Communications* (8.218) | Basic study | True | 28 | 1 | 12 |
| Structure-based classification of tauopathies | 150 | 142 | Shi, Y; Zhang, WJ; Yang, Y; Murzin, AG; Falcon, B; Kotecha, A; van Beers, M; Tarutani, A; Kametani, F; Garringer, HJ; Vidal, R; Hallinan, GI; Lashley, T; Saito, Y; Murayama, S; Yoshida, M; Tanaka, H; Kakita, A; Ikeuchi, T; Robinson, AC; Mann, DMA; Kovacs, GG; Revesz, T; Ghetti, B; Hasegawa, M; Goedert, M; Scheres, SHW | MRC Laboratory Molecular Biology; Thermo Fisher Scientific; Tokyo Metropolitan Institute of Medical Science; Indiana University System; Indiana University Bloomington; University of London; University College London; Tokyo Metropolitan Institute of Gerontology;  Osaka University; Aichi Medical University; Niigata University; Salford Royal NHS Foundation Trust; University of Manchester; University of Toronto; University of Toronto; Medical University of Vienna | *Nature* (63.581) | Basic study | True | 6 | 0 | 207 |
| Chronic neurodegeneration after traumatic brain injury: Alzheimer disease, chronic traumatic encephalopathy, or persistent neuroinflammation? | 143 | 81 | Faden, AI; Loane, DJ | University System of Maryland; University of Maryland Baltimore | *Neurotherapeutics* (8.296) | Review | True | 5 | 4 | 14 |
| In vivo characterization of chronic traumatic encephalopathy using [F-18] FDDNP PET brain imaging | 136 | 148 | Barrio, JR; Small, GW; Wong, KP; Huang, SC; Liu, J; Merrill, DA; Giza, CC; Fitzsimmons, RP; Omalu, B; Bailes, J; Kepe, V | University of California System; University of California Los Angeles; University of California Los Angeles Medical Center; David Geffen School of Medicine at UCLA; David Geffen School of Medicine at UCLA; University of California Davis; NorthShore University Health System; University of Chicago | *Proceedings of the National Academy of Sciences of the United States of America* (13.451) | Case control | True | 13 | 6 | 30 |
| Mixed pathologies including chronic traumatic encephalopathy account for dementia in retired association football (soccer) players | 134 | 1601 | Ling, HL; Morris, HR;  Neal, JW; Lees, AJ; Hardy, J; Holton, JL; Revesz, T; Williams, DDR | University of London; University College London; Cardiff University | *Acta Neuropathologica* (21.534) | Case series | True | 230 | 6 | 115 |
| Metabolic features of the cell danger response | 131 | 134 | Naviaux, RK | University of California System; University of California San Diego; | *Mitochondrion* (4.722) | Basic study | True | 6 | 5 | 112 |
| Chronic traumatic encephalopathy in sport: A systematic review | 121 | 104 | Gardner, A; Iverson, GL; McCrory, P | University of Newcastle; Harvard University; Harvard Medical School; Massachusetts General Hospital; Florey Institute of Neuroscience & Mental Health | *British Journal of Sports Medicine* (18.705) | Review | True | 6 | 2 | 55 |
| What is the evidence for chronic concussion-related changes in retired athletes: Behavioural, pathological, and clinical outcomes? | 108 | 115 | McCrory, P; Meeuwisse, WH; Kutcher, JS; Jordan, BD; Gardner, A | Florey Institute of Neuroscience & Mental Health; University of Calgary; University of Michigan System; University of Michigan | *British Journal of Sports Medicine* (18.705) | Review | True | 11 | 1 | 20 |
| Peripheral total tau in military personnel who sustain traumatic brain injuries during deployment | 107 | 87 | Olivera, A; Lejbman, N; Jeromin, A; French, LM; Kim, HS; Cashion, A; Mysliwiec, V; Diaz-Arrastia, R; Gill, J | National Institutes of Health (NIH) - USA;  NIH National Institute of Nursing Research (NINR); Walter Reed National Military Medical Center; Madigan Army Medical Center; Uniformed Services University of the Health Sciences - USA | *Jama Neurology* (22.581) | Cohort study | True | 7 | 0 | 62 |
| Tau prions from Alzheimer's disease and chronic traumatic encephalopathy patients propagate in cultured cells | 105 | 93 | Woerman, AL; Aoyagi, A; Patel, S; Kazmi, SA; Lobach, I; Grinberg, LT; Mckee, AC; Seeley, WW; Olson, SH; Prusiner, SB | University of California System; University of California San Francisco; Daiichi Sankyo Company Limited; Boston University; US Department of Veterans Affairs; Veterans Health Administration (VHA); Harvard University; VA Boston Healthcare System; | *Proceedings of the National Academy of Sciences of the United States of America* (13.451) | Basic study | True | 7 | 4 | 45 |
| Tau positron-emission tomography in former national football league players | 104 | 339 | Stern, RA; Adler, CH; Chen, KW; Navitsky, M; Luo, J; Dodick, DW; Alosco, ML; Tripodis, Y; Goradia, DD; Martin, B; Mastroeni, D; Fritts, NG; Jarnagin, J; Devous, MD; Mintun, MA; Pontecorvo, MJ; Shenton, ME; Reiman, EM | Boston University; Harvard University; Brigham & Women's Hospital; Harvard Medical School;  US Department of Veterans Affairs; Veterans Health Administration (VHA); VA Boston Healthcare System; Mayo Clinic; Mayo Clinic Phoenix; Banner Research; Banner Health; Banner Alzheimer's Institute; Arizona State University; Arizona State University-Tempe | *New England Journal of Medicine* (125.116) | Cohort | True | 26 | 8 | 265 |
| Chronic traumatic encephalopathy in contact sports: A systematic review of all reported pathological cases | 103 | 130 | Maroon, JC; Winkelman, R;  Bost, J; Amos, A; Mathyssek, C; Miele, V | Pennsylvania Commonwealth System of Higher Education (PCSHE); University of Pittsburgh | *Plos One* (4.069) | Review | True | 7 | 6 | 38 |
| Concussion in chronic traumatic encephalopathy | 103 | 105 | Stein, TD; Alvarez, VE; McKee, AC | US Department of Veterans Affairs; Veterans Health Administration (VHA); Harvard University; VA Boston Healthcare System; Boston University; | *Current Pain and Headache Reports* (4.11) | Review | True | 9 | 2 | 32 |
| Preliminary study of plasma exosomal tau as a potential biomarker for chronic traumatic encephalopathy | 100 | 516 | Stern, RA; Tripodis, Y; Baugh, CM; Fritts, NG; Martin, BM; Chaisson, C; Cantu, RC; Joyce, JA; Shah, S; Ikezu, T; Zhang, J; Gercel-Taylor, C; Taylor, DD | Boston University; University of Washington; Harvard University | *Journal of Alzheimers Disease* (5.279) | Case control | True | 104 | 0 | 6 |
| Age of first exposure to tackle football and chronic traumatic encephalopathy | 95 | 730 | Alosco, ML; Mez, J; Tripodis, Y; Kiernan, PT; Abdolmohammadi, B; Murphy, L; Kowall, NW; Stein, TD; Huber, BR; Goldstein, LE; Cantu, RC; Katz, DI; Chaisson, CE; Martin, B; Solomon, TM; McClean, MD; Daneshvar, DH; Nowinski, CJ; Stern, RA; McKee, AC | Boston University; Midwestern University;  US Department of Veterans Affairs; Veterans Health Administration (VHA); Harvard University;  VA Boston Healthcare System; US Department of Veterans Affairs; | *Annals of Neurology* (12.344) | Cohort | True | 89 | 3 | 91 |
| Chronic traumatic encephalopathy: The neuropathological legacy of traumatic brain injury | 90 | 126 | Hay, J; Johnson, VE; Smith, DH; Stewart, W | University of Glasgow; Queen Elizabeth University Hospital (QEUH); University of Pennsylvania; Pennsylvania Medicine; | *Annual Review of Pathology* (29.535) | Review | True | 9 | 2 | 148 |
